# Sensitive and Selective Electrochemical Detection of Lead(II) Based on Waste-Biomass-Derived Carbon Quantum Dots@Zeolitic Imidazolate Framework-8

**DOI:** 10.3390/ma16093378

**Published:** 2023-04-26

**Authors:** Qing Liu, Xiang Gao, Zhibao Liu, Ligang Gai, Yan Yue, Hongfang Ma

**Affiliations:** 1 School of Environmental Science and Engineering, Qilu University of Technology (Shandong Academy of Sciences), Jinan 250353, China; 2 Engineering and Technology Center of Electrochemistry, School of Chemistry and Engineering, Qilu University of Technology (Shandong Academy of Sciences), Jinan 250353, China

**Keywords:** carbon quantum dots, electrochemical sensor, heavy metal ions, wastes biomass

## Abstract

An electrochemical sensor based on carbon quantum dots (CQDs) and zeolitic imidazolate framework-8 (ZIF-8) composite was fabricated to detect lead(II). The CQDs (2.47 ± 0.52 nm) were synthesized from platanus acerifoli leaves by carbonization and the hydrothermal method. Under the optimal conditions, the fabricated electrochemical sensor had excellent performance in detecting Pb^2+^. The linear range for Pb^2+^ was 1 nM–1 μM, and the limit of detection (LOD) was 0.04 nM and the limit of quantification (LOQ) was 0.14 nM. Moreover, when the solution contained Pb^2+^ and Cd^2+^, the linear range for Pb^2+^ was 50 nM to 1 μM and the LOD was 0.02 nM. When the solution contained Pb^2+^ and Cu^2+^, the linear range for Pb^2+^ was 50 nM–750 nM and LOD was 0.07 nM. Furthermore, even if the solution contained Pb^2+^, Cd^2+^ and Cu^2+^, the linear range for Pb^2+^ was 50 nM–1 μM and the LOD was 0.04 nM. The X-ray photoelectron spectroscopy (XPS), Fourier transform infrared spectrometer (FTIR) and Brunauer-Emmet-Teller (BET) results indicated that the composite electrode materials had abundant oxygen-containing functional groups, a large specific surface area and pore structure, which are conducive to the adsorption of heavy metal ions and improve the detection performance.

## 1. Introduction

Heavy metals refer to metals with a density greater than 4.5 g/cm^3^, such as mercury(Hg), cadmium(Cd), lead(Pb) and chromium(Cr). The continuous development of industrialization and the increase in waste discharge have led to the aggravation of heavy metal pollution. Heavy metals are difficult to be biodegraded and causes serious harm to the environment. Moreover, the bio-magnification of the food chain can cause the accumulation of heavy metals in the human body, which can induce acute or chronic toxicity and various diseases, and seriously affect human health. As such, lead(II) is usually present in waste water produced from ore smelting, coal combustion, automotive exhausts, metal electroplating and more. The widespread use of lead has led to some environmental issues and related human health risks, such as excessive lead or lead poisoning that may cause damage to organs and nerves [1]. Therefore, the rapid and real-time detection of the concentration of heavy metal ions in the environment is of great significance for the prevention of heavy metal pollution.

At present, the commonly used methods for the detection of heavy metals include the terahertz (THz) spectrum, fluorescence detection and atomic absorption spectrophotometry [2,3,4]. These methods have high sensitivity and selectivity, but the sample pretreatment is complex, the detection cycle is long and it is not easy to carry out on-site detection. Electrochemical sensors have become a research hotspot because of their high sensitivity, strong selectivity and high efficiency.

The composition and structure of electrode materials determine the performance of the electrochemical sensor. To date, various functional materials such as carbon-based nanomaterials [5], metal oxides [6] and metal organic frameworks materials [7,8] have been used to modify the electrodes of electrochemical sensors to improve the performance. Carbon quantum dots (CQDs) are conjugated systems with a large number of sp^2^ hybrid orbitals, which can accelerate electron migration and have the functions of both electron transport and electron acceptors. Meanwhile, CQDs contain a large number of oxygen-containing functional groups on the surface, such as hydroxyl and carboxyl. CQDs are considered to be good materials for electrochemical sensors. Wang et al. [9] prepared composite materials of CQDs-doped tungsten disulfide and applied it to the detection of sulfamethazine. Wang et al. [10] synthesized CQDs for the rapid detection of Fe^3+^; the limit of detection (LOD) value was calculated to be 1.13 μM. However, CQDs have small specific surface areas and lack the ability of pre-concentration for trace pollutants, which impairs their detection sensitivity. It is an effective way to improve the sensitivity of electrochemical detection by combining it with other materials that have large specific surface areas and porous structures.

Metal organic frameworks (MOFs) materials are a kind of representative new multifunctional framework material [11,12]. MOFs have a large specific surface area and high porosity, which can provide more active adsorption sites for the electrochemical detection of heavy metals [13,14]. However, the poor conductivity of MOFs limit their application in electrochemical sensors. The incorporation CQDs with MOFs to prepare composites with good conductivity, rich oxygen-containing functional groups and a rich pore structure is expected to prepare electrochemical sensing electrodes with high sensitivity.

In this study, CQDs were synthesized from the waste biomass of platanus acerifoli leaves by carbonization at high temperature and then hydrothermal reaction with hydrogen peroxide. Then, CQDs were successfully modified on zeolitic imidazolate framework-8 (ZIF-8) using the simple ultrasonic method to prepare the composite carbon quantum dots@zeolitic imidazolate framework-8 (CQDs@ZIF-8). The properties of the synthesized materials were tested by electrochemical methods, including cyclic voltammetry (CV) and electrochemical impedance spectroscopy (EIS). Finally, the composite materials were applied to the electrochemical detection of Pb^2+^, Cd^2+^ and Cu^2+^ by differential pulse anodic stripping voltammetry (DPASV) measurement. The results showed that the composite materials could effectively detect heavy metals.

## 2. Materials and Methods

### 2.1. Materials

2-Methylimidazole and CdN_2_O_6_·4H_2_O were purchased from Shanghai Macklin Biochemical Co., Ltd., Shanghai, China, and zinc acetate dihydrate was purchased from Xilong Science Co., Ltd., Shantou, China. Methanol, anhydrous ethanol, H_2_O_2_ (30%), glacial acetic acid, CuSO_4_·5H_2_O, CdN_2_O_6_·4H_2_O, K_3_Fe(CN)_5_, KCl and CaCl_2_·2H_2_O were provided by Sinopharm Chemical Reagents Co., Ltd., Shanghai, China. PbCl_2_ was purchased from Shanghai Aladdin Biochemical Technology Co., Ltd., Shanghai, China. C_2_H_3_NaO_2_·3H_2_O and MgSO_4_ were provided by Tianjin Bodi Chemical Co., Ltd., Tianjin, China.

### 2.2. Synthesis of CQDs

The platanus acerifoli leaves were collected, washed and dried. The leaves were mixed with acetic acid solution, and reacted at 423 K for 3 h. After that, the leaves with the acid leaching reaction were washed with deionized water to neutral. They were then dried and ground into powder. Then, these powders were heated under N_2_ flow to obtain leaves-derived biochar. The reaction conditions are shown in Table S1.

**Figure 1:**
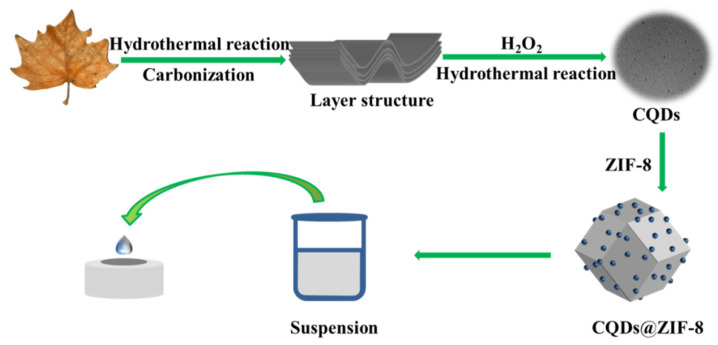
Diagram of preparation of carbon quantum dots@zeolitic imidazolate framework-8 (CQDs@ZIF-8).

As shown in Figure 1, CQDs were fabricated using the hydrothermal method [15]. An amount of 0.3 g leaves-derived biochar was dispersed into 20 mL H_2_O_2_ with magnetic stirring for 10 min and the mixture was heated at 493 K for 8 h. Lastly, the solution was filtered with an aperture of 0.22 μm to remove the insoluble substance.

### 2.3. Synthesis of ZIF-8

ZIF-8 was fabricated using the hydrothermal method. Amounts of 0.44 g of zinc acetate and 4.1 g of 2-methylimidazole were dissolved in 30 mL deionization water, respectively. Then, the two solutions were mixed under the condition of stirring. Later, the mixture was heated at 423 K for 6 h in an autoclave [16,17]. Finally, the product was collected by centrifugation, washed with water and ethanol and the sample was freeze-dried to obtain ZIF-8.

### 2.4. Synthesis of CQDs@ZIF-8

An amount of 0.1 g ZIF-8 was dispersed into 10 mL methanol and the solution was dispersed by ultrasound for 10 min. Then, CQDs solution was added and stirred for 30 min. Finally, the product was washed with methanol three times, and then dried at 333 K.

### 2.5. Electrode Preparation

A glassy carbon electrode (GCE) was polished with 0.05 μm alumina powder and cleaned by ultrasonication in water. The CQDs@ZIF-8, CQDs and ZIF-8 were respectively dispersed in alcohol with a concentration of 1 mg/mL. Then, 20 μL of dispersion was dropped onto the surface of the GCE and then dried under an infrared lamp (Figure 1).

### 2.6. Characterization Methods

The microstructure of the materials was investigated by scanning electron (SEM, Regulus 8100, Tokyo, Japan) and transmission electron (TEM, Tecnai G2, Portland, OR, USA) microscopes. Nitrogen adsorption/desorption isotherms were measured by a gas sorption analyzer (Autosorb-iQ, Boynton Beach, FL, USA) to confirm the pore structure and specific surface area of the materials. X-ray photoelectron spectroscopy (XPS) (Escalab 250Xi, Waltham, MA, USA), Fourier transform infrared (FTIR) spectra (IRAffinity-1s, Tokyo, Japan) and the diffraction of X-rays (XRD) (D8 advance, Karlsruhe, Germany) were used to confirm the chemical compositions, crystal structure and valence state of elements of the synthesized samples. The UV visible absorption effect of CQDs was obtained by UV-Vis spectroscopy (TU-1950, Beijing, China). Atomic absorption spectroscopy (AAS) (iCE 3000, Thermo, Waltham, MA, USA) was used to confirm the concentration of Pb^2+^ in real water samples. All the electrochemical measurements were carried out with a three-electrode system using a CHI electrochemical workstation (CHI760E, Shanghai, China). The three-electrode cell contained an Ag/AgCl reference electrode, a platinum auxiliary electrode and a GCE working electrode. CV and EIS measurements were conducted in 0.1 M KCl solution containing 5.0 mM K_3_[Fe(CN_6_)]. The used excitation potential was 5 mV, and the frequency range was from 100,000 Hz to 0.01 Hz during the EIS test. All electrochemical detections of heavy metals were carried out in acetic acid and sodium acetate buffer solution (HAc-NaAc, pH = 5).

## 3. Results and Discussion

### 3.1. Material Characterization

**Figure 2:**
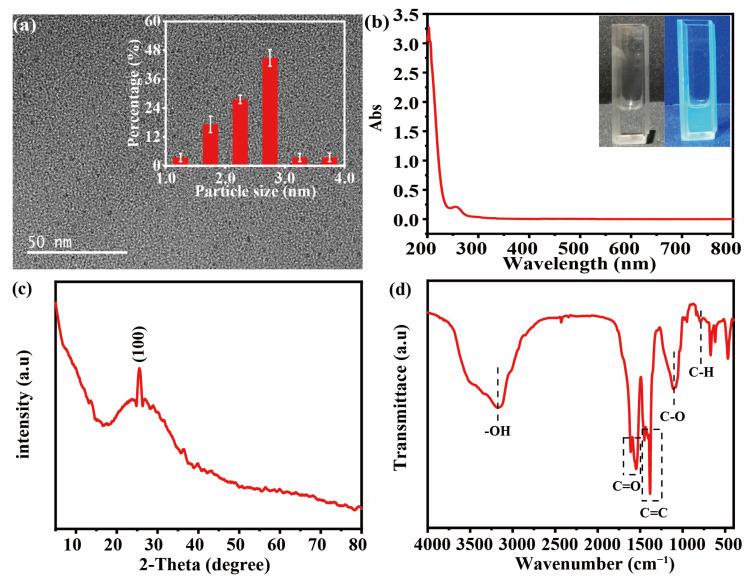
(**a**) HR-TEM images (Inset: Size distribution of CQDs), (**b**) UV-Vis absorbance spectrum (Inset: Photographs of CQDs under daylight (left) and 365 nm ultraviolet and visible (UV) light (right)), (**c**) XRD pattern and (**d**) FTIR spectra of CQDs.

TEM, UV-Vis and FTIR were used to investigate the structure and chemical composition of the synthesized CODs. As shown in Figure 2a, the synthesized CQDs were spherical particles with an average diameter of 2.47 ± 0.52 nm. The peak at 256 nm on the UV-vis spectrum shown in Figure 2b originates from the π-π* transition of C=C [18]. The existence of the π-π* transition can accelerate electron migration, which could improve the sensitivity of the electrochemical detection signal. Meanwhile, under the excitation of ultraviolet light, CQDs thread blue fluorescence effect. As shown in Figure 2c, in the XRD spectrum, the peak located at 25.5°correspond to the (100) plane of graphitic carbon. In Figure 2d, the peaks located at 3172 and 950 cm^−1^ originate from -OH stretching, the peaks centered at 1611 and 1548 cm^−1^ are designated to C=O stretching vibration and those at 1450 and 1383 cm^−1^ originate from C=C sp^2^ bending; the peaks centered at 1110 and 719 cm^−1^ are ascribed to C-O stretching and the external bending vibration of the aromatic C-H plane, respectively [19]. All of the results indicated that the CQDs were successfully synthesized from platanus acerifoli leaves.

**Figure 3:**
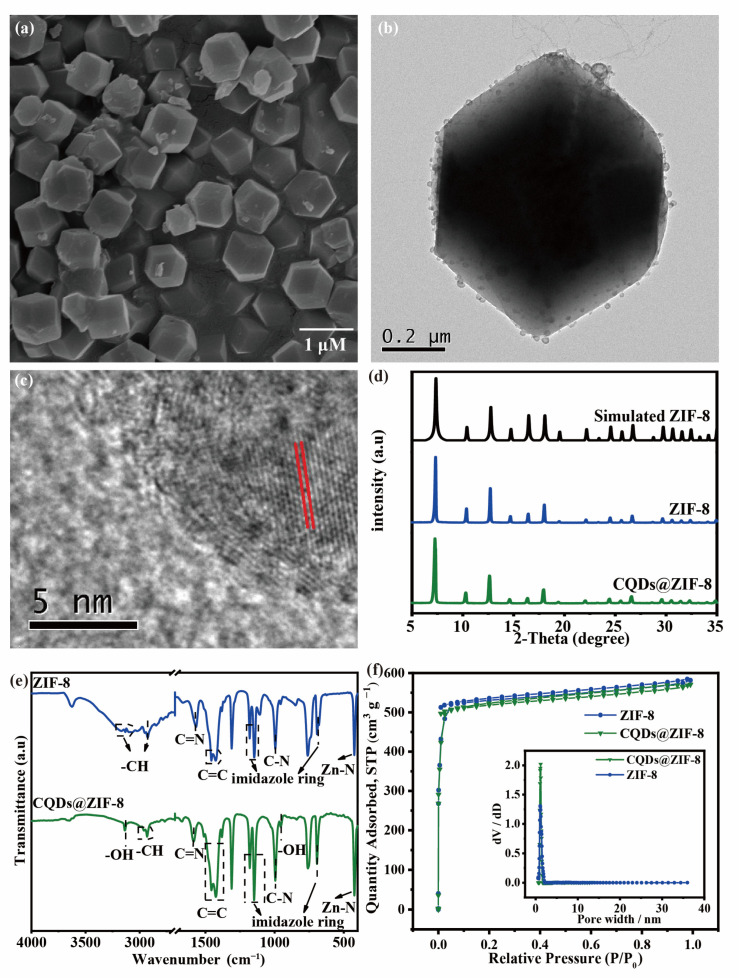
(**a**) TEM images of ZIF-8; (**b**) SEM and (**c**) HR-TEM images of CQDs@ZIF-8; (**d**) XRD pattern of Simulated ZIF-8, ZIF-8 and CQDs@ZIF-8; (**e**) FTIR spectra and (**f**) N_2_ adsorption/desorption isotherms (Inset: Pore size distribution) of ZIF-8 and CQDs@ZIF-8.

SEM, XRD, BET and FTIR were performed to identify the structure and composition of the synthesized ZIF-8. As shown in Figure 3a, the as-prepared ZIF-8 sample is a rhombic dodecahedron structure with a side length of about 500 nm. The XRD pattern shown in Figure 3d also demonstrates that the synthesized ZIF-8 exhibited a typical regular dodecahedral structure, which is basically identical to the simulated ZIF-8 structure. The peaks located at 7.32°, 10.36°, 12.7°, 14.68°, 16.44°, 18.02°, 24.5° and 26.66° corresponded to the (011), (002), (112), (022), (013), (222), (233) and (134) crystal planes of ZIF-8 [20]. The FTIR spectrum of the synthesized ZIF-8 shown in Figure 3e exhibited the characteristic peaks of ZIF-8. The peak at 424 cm^−1^ is the stretching vibration of Zn-N, the peak at 693 cm^−1^ is the out-of-plane bending vibration of the imidazole ring, the peak at 995 cm^−1^ corresponds to the stretching vibration of C-N, the peaks at 1147–1213 cm^−1^ are related to the bending of the imidazole ring, the peaks at 1427 and 1457 cm^−1^ are the stretching vibration of C=C, the peak at 1568 cm^−1^ is related to C=N, the peak at 2925 cm^−1^ is the irregular vibration of C-H in the fat ring and the peak near 3100 cm^−1^ is related to the C-H vibration of the aromatic [21]. All of the results demonstrated that the structure and composition of the synthesized ZIF-8 were consistent with those of the published literature. The N_2_ adsorption-desorption isotherm method was performed and the results are shown in Figure 3f; the corresponding factors obtained from the curves are shown in Table S2. The profile of the isotherms corresponds to that of type I, which is characteristic of microporous materials [22]. The pore size distribution indicates that ZIF-8 is composed of a large number of micropores, as shown in the inset of Figure 3f. This characteristic corresponds to the average pore diameter of ZIF-8, which is 1.645 nm in Table S2; furthermore, the BET surface area (S_BET_) and the total pore volume (V_tot_) of the ZIF-8 are 2186.9 m^2^/g and 0.900 cm^3^/g, respectively. The large specific surface area and pore volume are conducive to the adsorption of metal ions, thus improving the detection limit of heavy metal ions by electrochemical sensors. Herein, the combination of CQDs and ZIF-8 is to combine the advantages of both to prepare a composite electrode material (CQDs@ZIF-8).

TEM, XRD, FTIR and an N_2_ adsorption-desorption isotherm were used to confirm the structure and composition of CQDs@ZIF-8. As shown in Figure 3b, CQDs successfully loaded onto the ZIF-8 surface. The size of the loaded CQDs was larger than that shown in Figure 2. This may be due to the agglomeration of CQDs during the drying progress of the sample preparation. The HR-TEM images (Figure 3c) exhibit a crystalline structure with a lattice spacing of 0.23 nm, which relates to the (100) plane of graphitic carbon [23]. As shown in the XRD (Figure 3d), the diffraction peak of the composite materials is the same as that of ZIF-8. The reason that there is no diffraction peak observed for CQDs may be due to the low crystallinity of CQDs. The FT-IR spectra of CQDs@ZIF-8 also exhibit the characteristic peaks of CODs and ZIF-8 (Figure 3e). The peaks located at 423, 693, 1148, 1179, 1586, 1672, 2961 and 2931 cm^−1^ originate from ZIF-8. In general, it can be proven that the crystal structure of ZIF-8 in the composite has not been damaged and the original skeleton of ZIF-8 is preserved. Furthermore, the peak of the C=C stretching vibration of CQDs was at 841 cm^−1^, the peak of the -OH stretching vibration was located at 1026 cm^−1^ and the peak of the -OH stretching vibration in carboxylic acid was at 957 cm^−1^. All of the above results indicated that CQDs were successfully compounded on ZIF-8. The N_2_ adsorption-desorption isotherm indicated that the BET surface area (S_BET_) and the total pore volume (V_tot_) of the CQDs@ZIF-8 were 2162.1 m^2^/g and 0.883 cm^3^/g, respectively. Compared with ZIF-8, there was almost no change in pore size distribution, and the average pore diameter was 1.63 nm. It proves that CQDs@ZIF-8 was still a microporous material, together with Figure 3f. The specific surface area of CQDs@ZIF-8 was smaller than ZIF-8 because of the aggregation of CQDs on the surface of ZIF-8. However, the composite still had a large specific surface area, which provides a large number of active sites for the adsorption of heavy metals.

**Figure 4:**
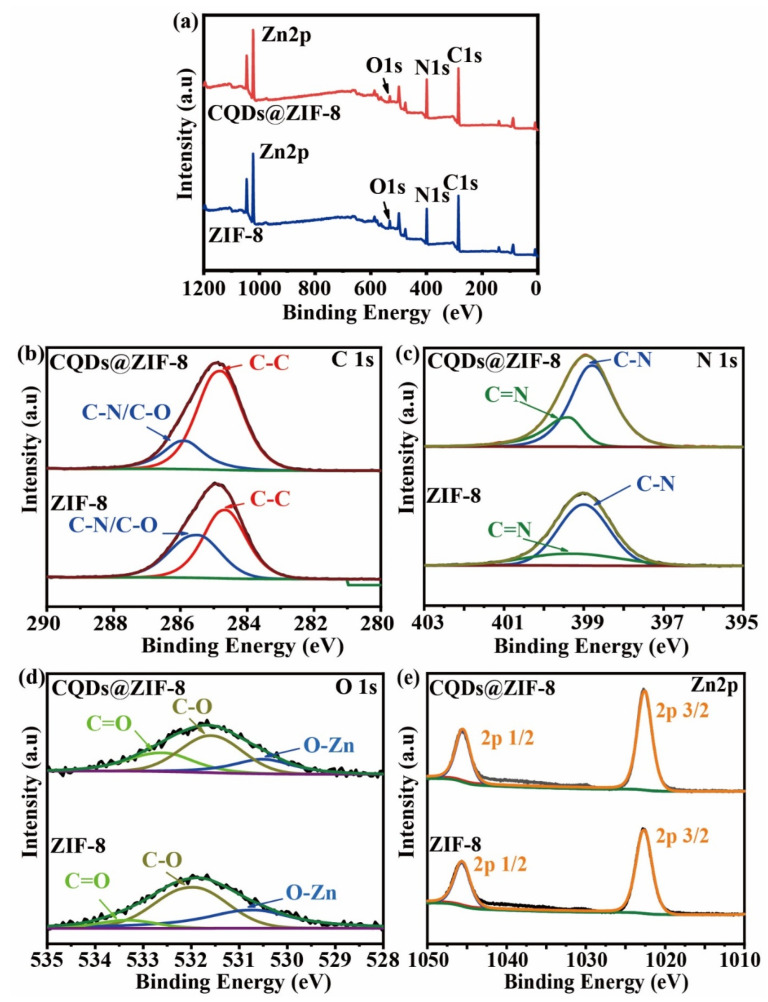
(**a**) XPS survey spectra of ZIF-8 and CQDs@ZIF-8; (**b**) C1s, (**c**) N1s, (**d**) O1s and (**e**) Zn2p of ZIF-8 and CQDs@ZIF-8.

XPS was conducted to analyze the chemical states of the elements in the ZIF-8 and CQDs@ZIF-8 composite (Figure 4). In the C1s spectra (Figure 4b), the peaks of ZIF-8 at 284.7 and 285.5 eV are ascribed to C-C and C-N/C-O, and the C 1s for CQDs@ZIF-8 could also be deconvoluted into two peaks corresponding to C-C and C-N/C-O with the binding energies at 284.4 and 285.9 eV, respectively [24]. The N1s (Figure 4c) for ZIF-8 has two peaks at 398.8 and 399.3 eV, which are assigned to C-N and C=N in 2-methylimidazole [25]. The peaks of N1s for CQDs@ZIF-8 hardly change compared with ZIF-8, which are located at 398.8 and 399.4 eV. The O1s of ZIF-8 (Figure 4d) is deconvoluted into three peaks, corresponding to O-Zn, C-O and C=O, with binding energies of 530.7, 532.0 and 533.3 eV, respectively, while the positions of the three peaks for CQD@ZIF-8 are located at 530.5, 532.7 and 533.8 eV. According to the peak areas of C=O for ZIF-8 and CQD@ZIF-8, the ratio of C=O in CQD@ZIF-8 is much higher than that in ZIF-8. This may be due to the fact that CQDs contain abundant oxygen-containing functional groups. As shown in Figure 4e, the Zn 2p spectra of ZIF-8 and CQDs@ZIF-8 at 1022.6 and 1045.6 eV are assigned to Zn 2p^3/2^ and Zn 2p^1/2^ hybrid orbitals [25]. According to the XPS results, the CQDs@ZIF-8 contains abundant oxygen-containing functional groups, which are beneficial to the enrichment of heavy metal ions.

### 3.2. Electrochemical Characteristics

CV curves and EIS spectra were used to evaluate the electrochemical behavior of the different electrodes in 0.1 M KCl solution with 5.0 mM K_3_[Fe(CN_6_)]. As shown in Figure S1a, the CV curves of the different electrodes shown in Figure S1a can reflect the electron transfer rate and the reversibility of each electrode. All of the curves contained a pair of almost symmetrical oxidation-reduction peaks. Compared with ZIF-8, the peak current of oxidation peak and reduction peak in CQDs@ZIF-8 increased, which indicated that the introduction of CQDs increases the electrical conductivity of the ZIF-8 electrode. The conductivity and interfacial behavior of the electrodes were also studied by EIS method. As shown in Figure S1b, all of the Nyquist plots consisted of one semi-circle in high frequency and one straight line in low frequency. The semi-circle in high frequency originated from the charge transfer process at the electrode/solution interface. The straight line in low frequency reflected the diffusion process. The EIS spectra were fitted by the equivalent circuit *R*_s_(*Q_dl_*(*R*_ct_*W*)). In the circuit, *R*_s_ was the solution resistance, *R*_ct_ was the charge transfer resistance, *Q_dl_* was the electrical double-layer capacitor and *W* reflected the Warburg resistance. As shown in Table 1, the *R*_ct_ values of the three modified GCE were all larger than that of bare GCE. This may because the ZIF-8 is one semiconductor and could not obviously improve the electrical conductivity of GCE. The *R*_ct_ value of CQDs@ZIF-8 was smaller than that of the ZIF-8 electrodes, which indicated that the CQDs could improve the electrical conductivity of ZIF-8. The *Y*_0_ values of the four electrodes revealed the same variation tendency with the *R*_ct_ values. These results are in agreement with the results of CV, indicating that the electrical conductivity of the ZIF-8-modified electrode was enhanced by combining CQDs. The effective surface area of the modified electrodes can be calculated by using the Randles-Sevcik equation [26]:

(1) $$\mathrm{Ip}=2{.69\;\times \;10}^{5}{\mathrm{AD}}^{1/2}n^{3/2}v^{1/2}C$$

where A is the effective surface area, D is the diffusion coefficient of K_3_[Fe(CN_6_)] (6.67 × 10^−6^ cm^2^s^−1^), n is the number of electrons transferred (n = 1), ν is the scan rates and C is the bulk concentration of K_3_[Fe(CN_6_)]. According to Figure S1 c–f, the effective surface area of GCE was calculated to be 0.147 cm^2^. The area of the CQDs@ZIF-8 electrode was 0.144 cm^2^.

**Figure 5:**
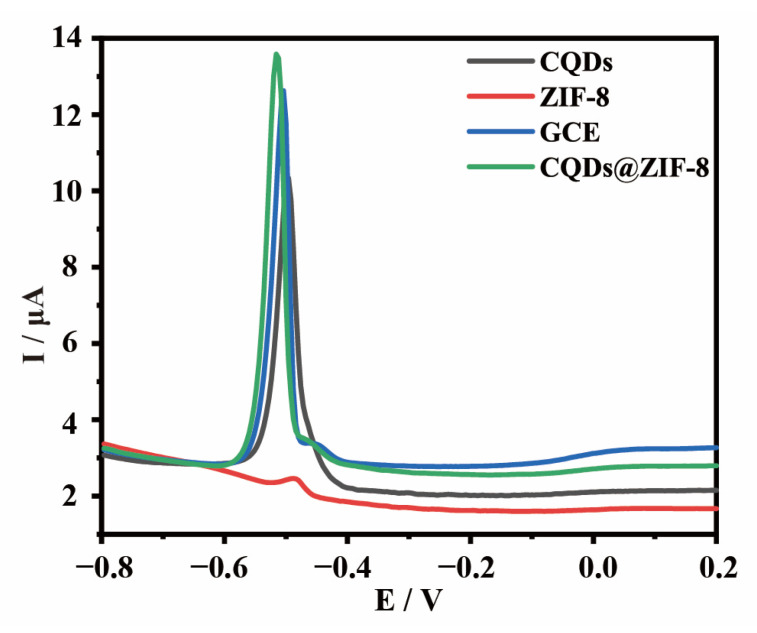
DPASV responses for bare and ZIF-8, CQDs and CQDs@ZIF-8 modified GCE in 1 μM Pb^2+^ solution.

In order to compare the detection performance of the different electrode materials, the four electrodes were used to detect 1 μM Pb^2+^ using the DPASV method. As shown in Figure 5, the peak current of CQDs@ZIF-8 is greater than that of other electrodes. These results indicate that the detection performance of CQDs@ZIF-8 is better than that of bare and ZIF-8, CQDs modified GCE.

### 3.3. Electrochemical Detection of Pb^2+^

**Figure 6:**
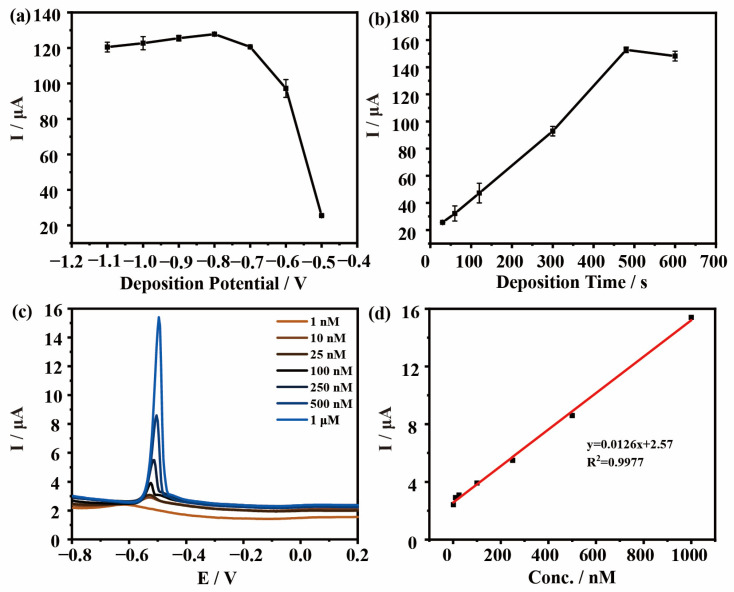
The effects of (**a**) deposition potential and (**b**) deposition time on the current response of CQDs@ ZIF-8; (**c**) DPASV responses of CQDs@ZIF-8 at various concentrations of Pb^2+^; (**d**) Linear correlation curves for Pb^2+^.

The deposition potential and time obviously affect the response of DPASV measurements. Therefore, different deposition potentials (from −0.5 V to −1.1 V) (Figure 6a) and deposition times (from 30 s to 480 s) (Figure 6b) were examined in order to achieve the best detection efficiency. As shown in Figure 6a, when the potential is −0.8 V, the current value is the largest, indicating that the detection signal is the strongest, so the optimal potential is −0.8 V. As the potential continues to increase, the corresponding current density decreases because water participates in the reaction to produce bubbles attached to the electrode surface, resulting in the reduction of heavy metal adsorption on the electrode surface [27]. In Figure 6b, the DPASV response to the pre-concentrated electrodes shows a steady increase in the stripping current for Pb^2+^ and the pre-concentration of Pb^2+^ over the electrode attains saturation after 480 s. When the time continues to increase, the peak current does not continue to increase, which is due to the saturation of the active site of the electrode materials and the inability to continue to deposit heavy metals. Therefore, the optimal conditions for the detection of Pb^2+^ were with a deposition voltage of −0.8 V and a deposition time of 480 s. Under these conditions, the peak current of Pb^2+^ linearly increased with an increase in concentration (Figure 6c). For Pb^2+^, an excellent linearity was presented in the concentration range of 1 nM–1 μM (Figure 6d). The limit of detection (LOD) and the limit of quantification (LOQ) are calculated by Equations (2) and (3) [28]:

(2) LOD=3σ/S

(3) LOQ=10σ/S

where σ is the standard deviation of 10 consecutive DPASV blanks and S is the calibration curve slope.

The LOD for Pb^2+^ of the CQDs@ZIF-8 electrode is 0.04 nM and the LOQ is 0.14 nM. Furthermore, the experiments for the detection of Pb^2+^ using the GCE and ZIF-8 and CQDs-modified electrode have a lower linear relationship (Figure S2).

**Figure 7:**
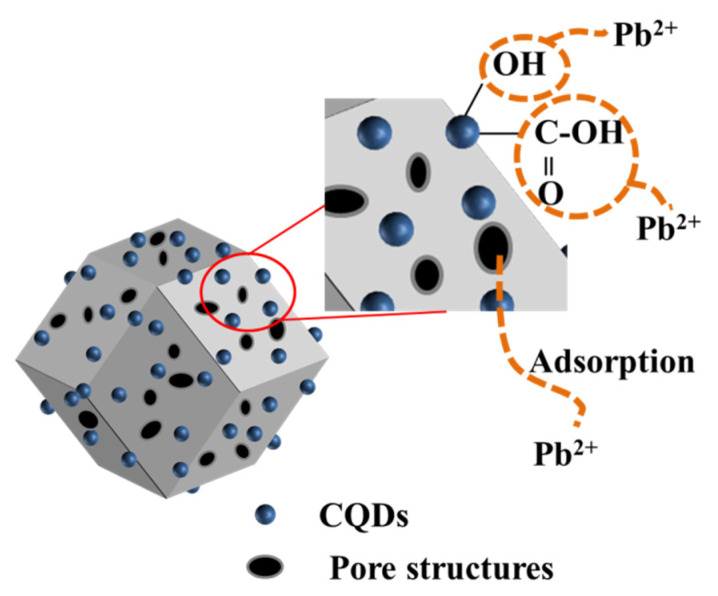
Electrochemical detection mechanism of CQDs@ZIF-8.

In general, the detection performance of CQDs@ZIF-8 is better than CQDs and ZIF-8. The reasons for these results are summarized in Figure 7. ZIF-8 has a large specific surface area and a rich pore structure, which provides a large number of active sites for the adsorption of heavy metals. Meanwhile, the surface of CQDs contains rich oxygen-containing functional groups such as -OH, -COOH and more, which assist the heavy metal ion adsorption properties and increase the redox ability. These characteristics make the electrochemical detection of composite materials more sensitive than that of single materials. As shown in Table 2, compared with other published work, the linear concentration range and the LOD for detecting Pb^2+^ of CQDs@ZIF-8 were both competitive. Meanwhile, the CODs were synthesized through platanus acerifoli leaves, which can achieve the resource utilization of waste biomass. So, the synthesized method and electrode materials both exhibited good application prospects.

### 3.4. Selectivity and Interference Measurements

**Figure 8:**
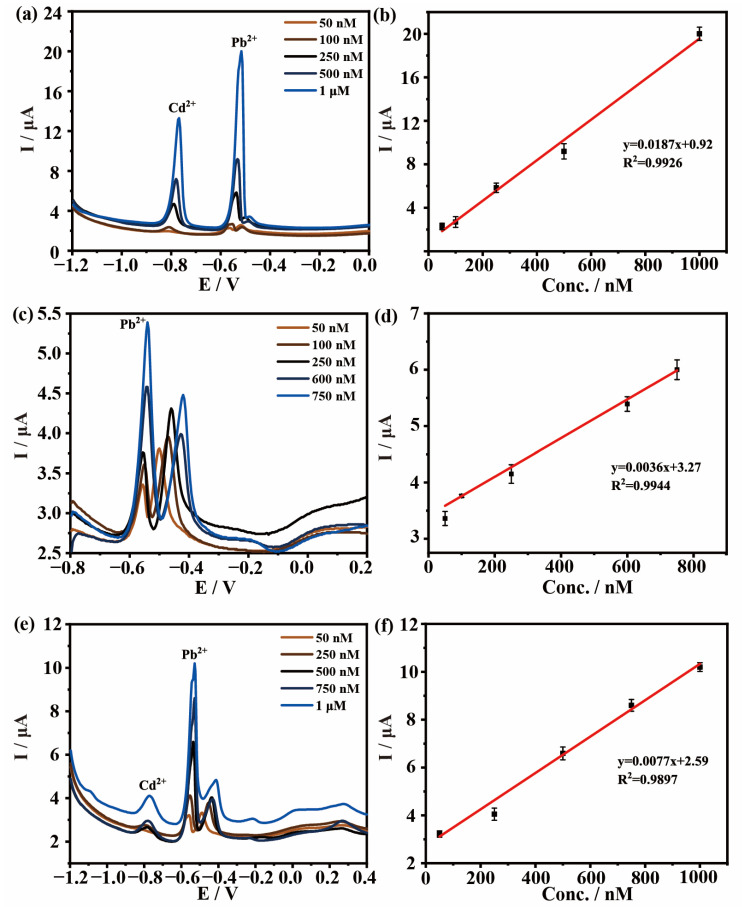
(**a**,**c**,**e**) DPASV responses and (**b**,**d**,**f**) linear correlation curves for Pb^2+^ at various concentrations of coexisting ions (**a**,**b**) Pb^2+^ and Cd^2+^, (**c**,**d**) Pb^2+^ and Cu^2+^ and (**e**,**f**) Pb^2+^, Cd^2+^ and Cu^2+^.

The interaction between different components will affect the sensitivity of electrochemical detection. The selectivity and interference of electrode materials were verified by adding Cd^2+^ and Cu^2+^. As observed in Figure S3, the stripping peak potential of Cd^2+^ and Cu^2+^ appeared at −0.75 V and 0 V, respectively. It indicates that Cd^2+^, Cu^2+^ and Pb^2+^ can be simultaneously detected due to separated stripping peaks. As shown in Figure 8, DPASV is used for the electrochemical detection of multiple heavy metals. Due to the difference in redox voltage between these three metal particles, we set the deposition voltage to −1.0 V in these experiments. Figure 8a,b shows the detection results of the mixed solution containing Pb^2+^ and Cd^2+^. It can be seen that there is a good linear relationship between the peak current and the concentration of Pb^2+^ when the concentrations of Pb^2+^ range from 50 nM to 1 μM. The LOD is 0.02 nM. Figure 6c,d shows the detection results of the mixed solution containing Pb^2+^ and Cu^2+^. There is a good linear relationship between the Pb^2+^ concentration and the peak current in the range of 50 nM–750 nM, and the LOD is 0.07 nM. Figure 6e,f shows the electrochemical detection of solutions containing Pb^2+^, Cd^2+^ and Cu^2+^. There is a good linear relationship between the concentration and peak current of Pb^2+^ in the range of 50 nM–1 μM and the LOD is 0.04 nM. Overall, the electrode of CQDs@ZIF-8 is capable of measuring the concentration of Pb^2+^ with coexisting ions. The detection limit is relatively low and the detection range is wide. These prove that the electrochemical detection selectivity of the composite materials is fine.

As shown in Figure S3, other heavy metal ions in the mixed solution could also be detected in quantification within a certain concentration range. When the solution contained Pb^2+^ and Cd^2+^, the detection range of Cd^2+^ was 50 nM–1 μM and *R*^2^ was 0.9981. When the solution contained Pb^2+^ and Cu^2+^, the detection range of Cu^2+^ was 10 μM–100 μM (*R*^2^ = 0.9891). In the mixed solution of Pb^2+^, Cd^2+^ and Cu^2+^, the detection range for Cd^2+^ was 1 μM–500 μM and *R*^2^ was 0.9959. Meanwhile, the detection range for Cu^2+^ was 0.75 μM–50 μM (*R*^2^ = 0.9844). All these results proved that CQDs@ZIF-8 is a highly selective electrode material.

**Figure 9:**
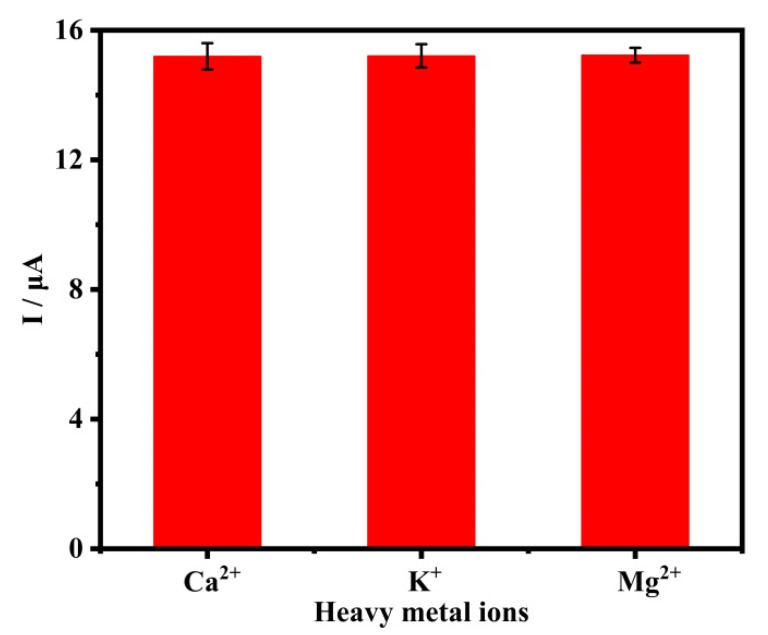
Interference study of fabricated CQDs@ZIF-8 sensors.

The anti-interference performance of CQDs@ZIF-8 is further verified by adding other ions, such as Ca^2+^, Mg^2+^ and K^+^, respectively. The concentration of these three ion concentrations is 5 μM, which is five times that of Pb^2+^ (1 μM). As shown in Figure 9, the electrochemical detection results of Pb^2+^ are basically unaffected by the added interfering ions, and the relative standard deviation (RSD) for Ca^2+^, Mg^2+^ and K^+^ are 2.68%, 1.48% and 2.37%, respectively. All these results prove that CQDs@ZIF-8 has a good performance in anti-interference.

### 3.5. Reproducibility and Stability Study

**Figure 10:**
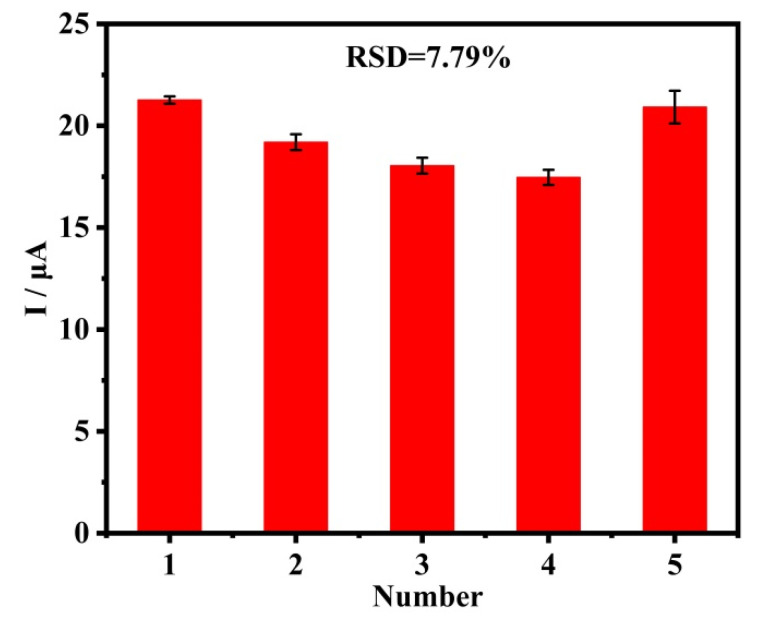
Reproducibility and stability measurements of five fabricated CQDs@ZIF-8 sensors in 1 μM Pb^2+^. Every sensor was continuously monitored five times.

Reproducibility and stability are prominent indicators for electrodes in commercial application. In order to evaluate the reproducibility and stability of the fabricated sensors, five parallel electrodes were used to detect the peak currents of Pb^2+^ under the same conditions. In order to evaluate the stability of the fabricated sensors, each sensor was continuously monitored five times. As shown in Figure 10, the DPASV responses slightly fluctuate and have a lower relative standard deviation (RSD), which is 7.79%. This proves that the CQDs@ZIF-8-modified electrode has good reproducibility and stability in the detection of heavy metals.

### 3.6. Analytical Application

In order to evaluate the performance of the electrode material, CQDs@ZIF-8 was applied to the detection of Pb^2+^ in real water samples. The real water samples were laboratory tap water, river water and industrial wastewater. All of the samples were filtered through a 0.22 μm membrane to remove the suspended solids. There was no response of Pb^2+^ for tap water and river water. It was indicated that almost no Pb^2+^ existed in tap water and river water. For industrial wastewater, the electrochemical detection results revealed that the concentration of Pb^2+^ was 278.57 ± 13.64 nM. In order to verify the accuracy of the results, the AAS method was used on the same samples. The standard curve of AAS for Pb^2+^ is shown in Figure S4. According to the AAS results, there was also no Pb^2+^ for tap water and river water, and the concentration of Pb^2+^ in industrial wastewater was 281.26 ± 1.18 nM. These results are basically consistent with the electrochemical detection results. The standard solution of Pb^2+^ was spiked into tap water and river water for recovery evaluation (repeated three times). As shown in Table 3, the recovery for the determination of Pb^2+^ in tap water and river water was 99.23% ± 6.47% and 95.44% ± 1.55%, respectively, indicating that CQDs@ZIF-8 could be used for the sensitive determination of Pb^2+^ in real water samples.

## 4. Conclusions

CQDs with an average diameter of 2.47 ± 0.52 nm derived from platanus acerifoli leaves were successfully synthesized. Furthermore, the composited electrode materials CQDs@ZIF-8 were fabricated by the synthesized CQDs and ZIF-8. The FTIR, XPS and BET results demonstrated that CQDs@ZIF-8 contained abundant oxygen-containing functional groups, and a high specific surface area. DPASV measurements indicated that CQDs@ZIF-8 exhibited high sensitivity and selectivity in the electrochemical detection of Pb^2+^. For the single solution, the linear range for Pb^2+^ was 1 nM–1 μM, the LOD was 0.04 nM and LOQ was 0.14 nM. Moreover, when the solution contained Pb^2+^ and Cd^2+^, the linear range for Pb^2+^ was 50 nM to 1 μM, and when the solution contained Pb^2+^ and Cu^2+^, the linear range for Pb^2+^ was 50 nM–750 nM. Furthermore, in the presence of Pb^2+^, Cu^2+^ and Cd^2+^, the linear range for Pb^2+^ was 50 nM–1 μM. Meanwhile, CQDs@ZIF-8 had good repeatability. High sensitivity and selectivity, together with favorable repeatability, stability and anti-interference ability, promote the application of CQDs@ZIF-8 in the electrochemical detection of heavy metal ions.

## Figures and Tables

**Table 1:** The fitting data of EIS for different materials.

Materials	*R*ct (Ω·cm^2^)	*R*s (Ω·cm^2^)	*Q*_dl_		*W* (Ω^−1^ cm^−2^s^n^)
			*Y*_0_ (Ω^−1^ cm^−2^s^n^)	*n*	
ZIF-8	6272	13.50	8.839 × 10^−5^	0.8402	3.207 × 10^−4^
CQDs	3681	12.59	2.791 × 10^−5^	0.8948	3.580 × 10^−4^
CQDs@ZIF-8	5192	13.53	5.236 × 10^−5^	0.8249	2.700 × 10^−4^
GCE	4052	13.41	3.358 × 10^−5^	0.8907	3.866 × 10^−4^

**Table 2:** Comparison with other published work for the detection of Pb^2+^.

Electrode	Method	Linear Concentration Range	LOD	References
l-Arginine-RGO ^1^/GCE	DPASV ^2^	1–1000 nM	0.06 nM	[29]
AuNFs ^3^/Y-DNA	EIS ^4^	0.5–1000 nM	0.38 nM	[30]
GO ^5^-imi-(CH_2_)_2_-NH_2_/CPE ^6^	DPASV	5.0–300.0 nM	0.30 nM	[31]
In-doped Bi_2_S_3_/GCE	SWASV ^7^	0.1–1.0 μM	0.017 μΜ	[32]
rGO/MWCNT ^8^/AuNP ^9^/GCE	DPV ^10^	0.05–200 nM	0.0071 nM	[33]
NiMn_2_O_4_-graphene/GCE	SWASV	1.4–7.7 μM	0.05 μM	[34]
Mercury films/paper electrodes	LSV ^11^	2.4–4.8 μM	0.48 μM	[35]
Bi/PXB ^12^/GCE	DPASV	19.3–530.9 nM	13.5 nM	[36]
CQDs@ZIF-8/GCE	DPASV	1–1000 nM	0.04 nM	This work

^1^ Reduced graphene oxide. ^2^ Differential pulse anodic stripping voltammetry. ^3^ Gold nanoflowers. ^4^ Electrochemical impedance spectroscopy. ^5^ Graphene oxide. ^6^ Carbon paste electrode. ^7^ Square-wave anodic stripping voltammetry. ^8^ Multiwalled carbon nanotubes. ^9^ Gold nanoparticles. ^10^ Differential pulse voltammetry. ^11^ Linear sweep voltammetry. ^12^ Poly (xylenol blue).

**Table 3:** Recoveries of heavy metal ion in water samples.

Samples	Add/nM	Found/nM	Recovery/%
Tap water	500	496.17 ± 32.38	99.23 ± 6.47
River water	500	477.20 ± 7.74	95.44 ± 1.55

## Data Availability

The data presented in this study are available on request from the corresponding author.

## Supplementary Materials

The following supporting information can be downloaded at: https://www.mdpi.com/article/10.3390/ma16093378/s1, Table S1: Heating process of preparing the biochar; Table S2: S_BET_, pore volume and average pore diameter of ZIF-8 and CQDs@ZIF-8; Figure S1. (a) CV (scan rate of 50 mV/s) for CQDs, ZIF-8 and CQDs@ZIF-8 and (b) EIS for CQDs, ZIF-8, CQDs@ZIF-8 and GCE; CV curves of (c) GCE and (e) CQDs@ZIF-8 at different scan rate (10, 20, 30, 40, 50, 60, 70, 80, 90, 100 mV/s) with the same solution; Relationship between peak current vs. square root of scan rate derived from the CV curves of (d) GCE and (f) CQDs@ ZIF-8; Figure S2: DPASV responses of (a) GCE and (c) CQDs at various concentrations of Pb^2+^; (b,d) linear correlation curves for Pb^2+^. Deposition potential: −0.8 V, Deposition time: 480 s; Figure S3: (a,c,e,g) DPASV responses and (b,d,f,h) linear correlation curves for (a,b,e,f) Cd^2+^ and (c,d,g,h) Cu^2+^ at various concentration coexisting ions (a,b) Pb^2+^ and Cd^2+^, (c,d) Pb^2+^ and Cu^2+^ and (e,f,g,h) Pb^2+^, Cd^2+^ and Cu^2+^; Figure S4. Standard curve of AAS for Pb^2+^.
