# Supplementary material for: Sensitive and Selective Electrochemical Detection of Lead(II) Based on Waste-Biomass-Derived Carbon Quantum Dots@Zeolitic Imidazolate Framework-8
Source: Materials (Basel). 2023 Apr 26;16(9):3378. doi: 10.3390/ma16093378 (PMC10180021; doi:10.3390/ma16093378)
Supplement: Supplementary file 1 [file materials-16-03378-s001.zip › materials-2328745-supplementary.pdf]

Supplementary Materials

Sensitive and Selective Electrochemical Detection of Lead(II) Based on Waste-Biomass-Derived Carbon Quantum Dots@Zeolitic Imidazolate Framework-8

Qing Liu[1], Xiang Gao[2], Zhibao Liu[2], Ligang Gai[2], Yan Yue[2], Hongfang Ma[1,*]

[1]School of Environmental Science and Engineering, Qilu University of Technology (Shandong Academy of Sciences), Jinan 250353, China.
[2]Engineering & Technology Center of Electrochemistry, School of Chemistry and Engineering, Qilu University of Technology (Shandong Academy of Sciences), Jinan 250353, China.
*Corresponding author: merryhf@163.com

Table S1. Heating process of preparing the biochar.

Heating rate	Final temperature	Retention time
283 K/min	773 K	3 h

Table S2. S_{BET}, pore volume and average pore diameter of ZIF-8 and CQDs@ZIF-8.

Sample	S_{BET}(m^2/g)	Pore volume (cm^3/g)	Average pore diameter (nm)
ZIF-8	2,186.9	0.900	1.645
CQDs@ZIF-8	2,162.1	0.883	1.633

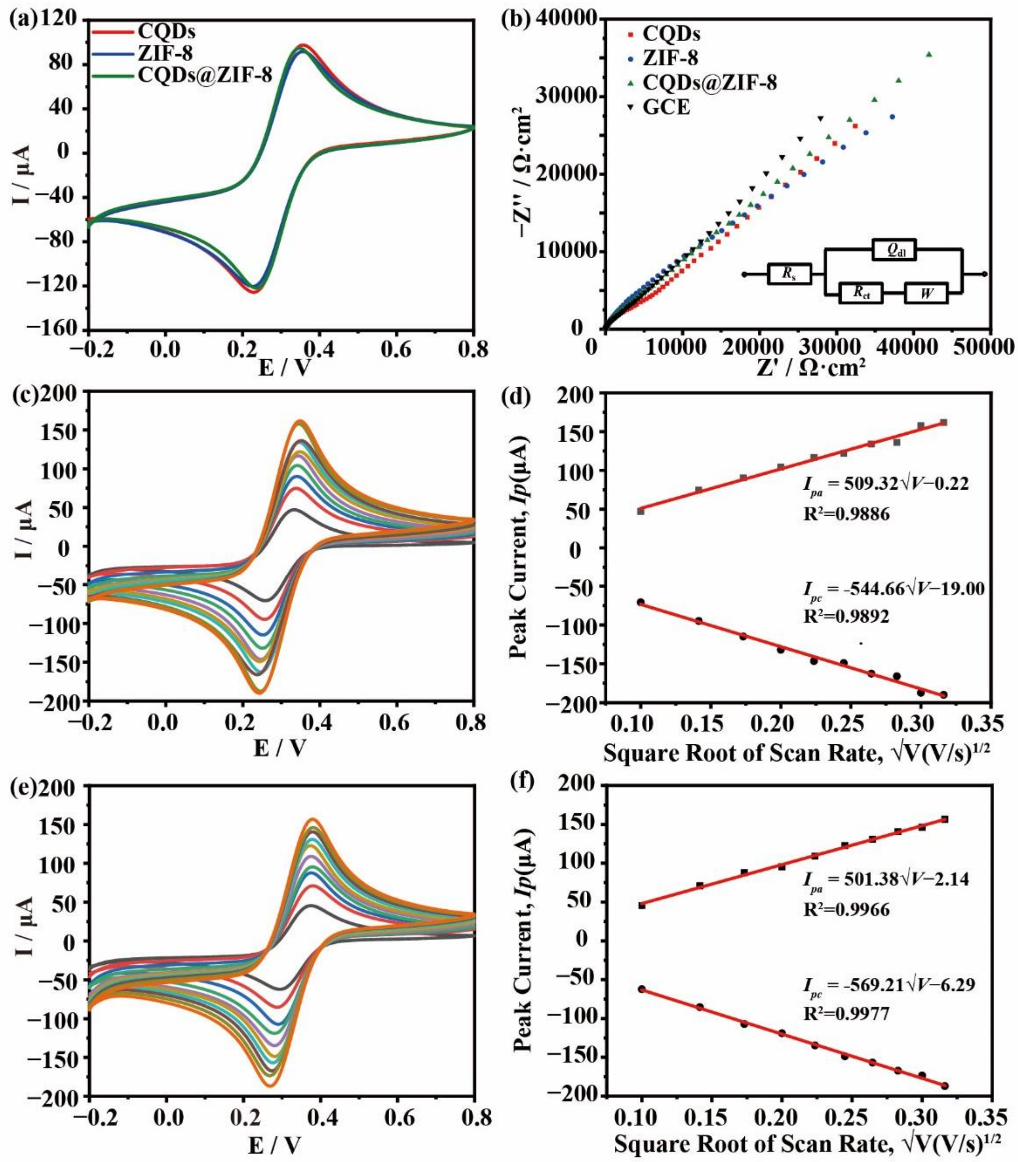

Figure S1. (**a**) CV (scan rate of 50 mV/s) for CQDs, ZIF-8 and CQDs@ZIF-8 and (**b**) EIS for CQDs, ZIF-8, CQDs@ZIF-8 and GCE; CV curves of (**c**) GCE and (**e**) CQDs@ZIF-8 at different scan rate (10, 20, 30, 40, 50, 60, 70, 80, 90, 100 mV/s) with the same solution; Relationship between peak current vs square root of scan rate derived from the CV curves of (**d**) GCE and (**f**) CQDs@ ZIF-8.

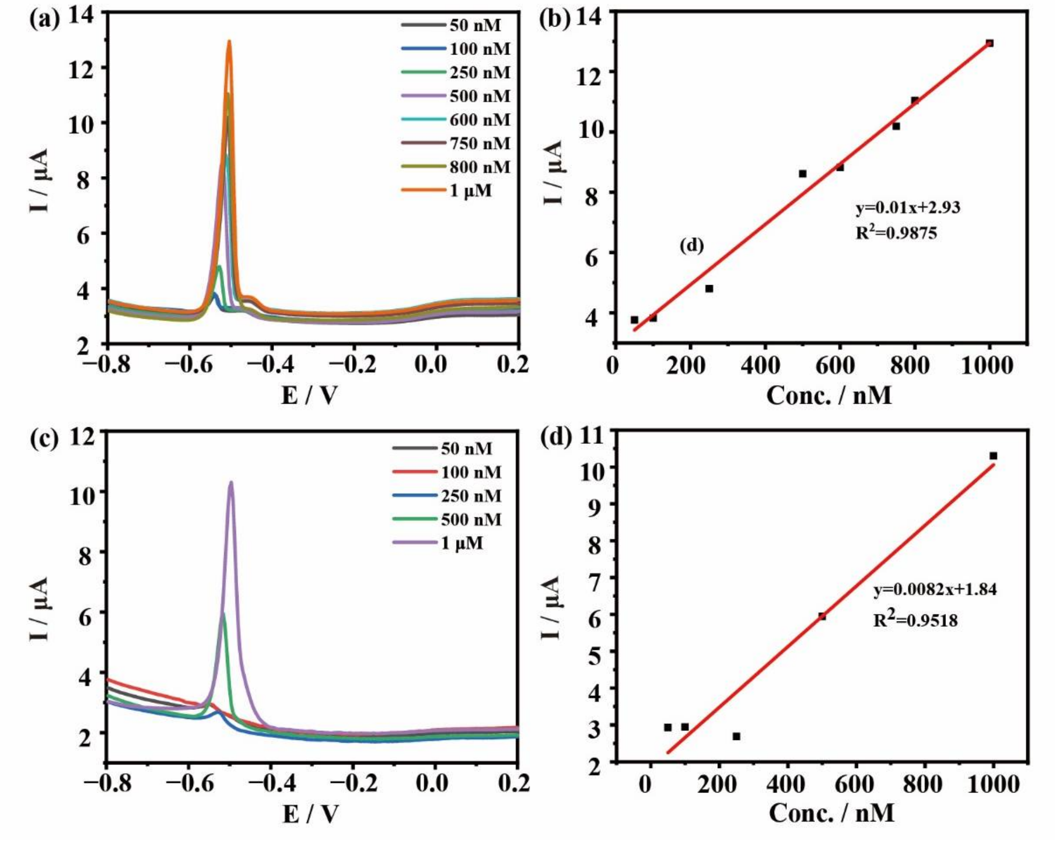

Figure S2. DPASV responses of (**a**) GCE and (**c**) CQDs at various concentrations of Pb^{2+}; (**b**, **d**) linear correlation curves for Pb^{2+}. Deposition potential: −0.8 V, Deposition time: 480 s.

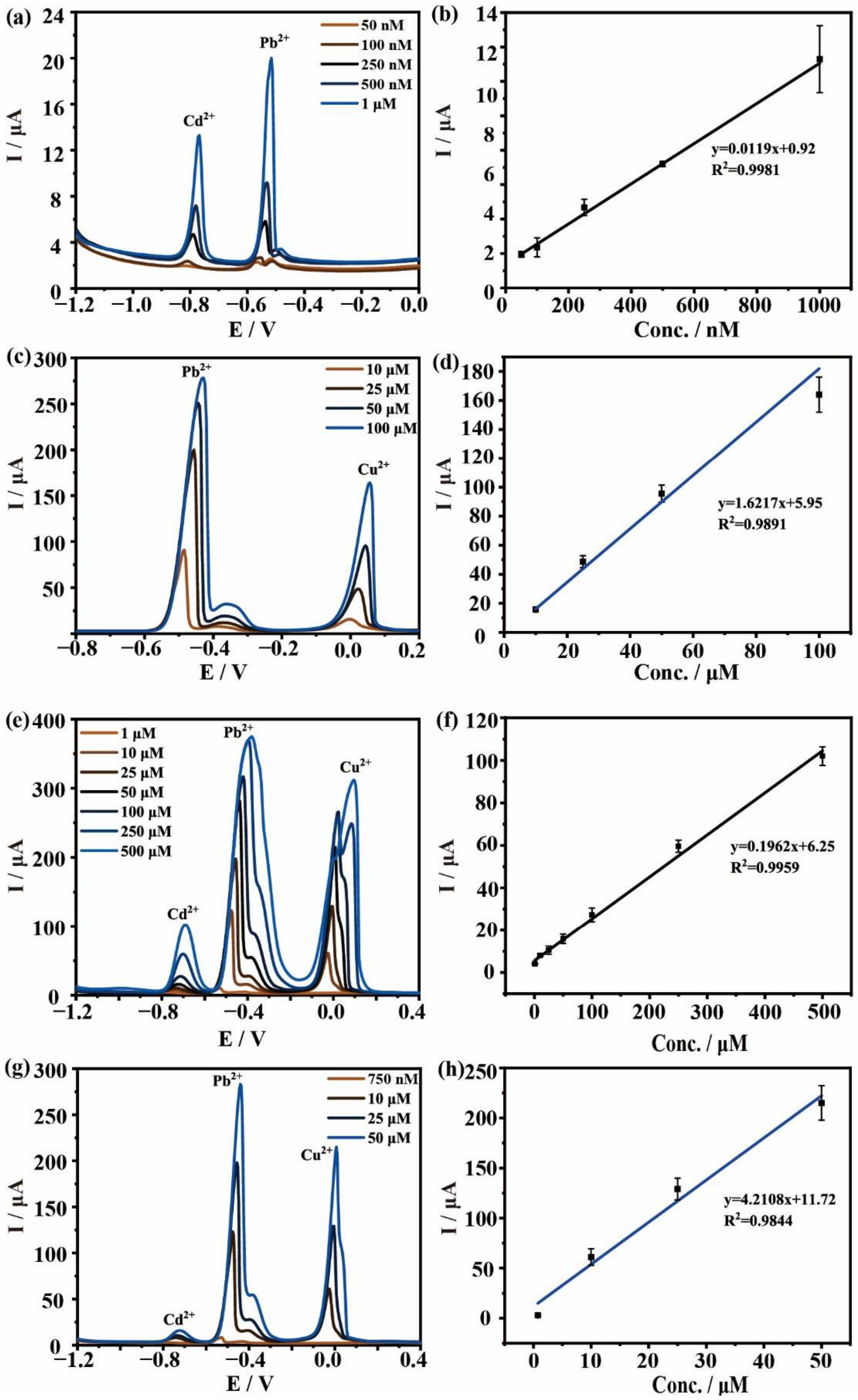

Figure S3. (**a**, **c**, **e**, **g**) DPASV responses and (**b**, **d**, **f**, **h**) linear correlation curves for (**a**, **b**, **e**, **f**) Cd^{2+} and (**c**, **d**, **g**, **h**) Cu^{2+} at various concentration coexisting ions (**a**, **b**) Pb^{2+} and Cd^{2+}, (**c**, **d**) Pb^{2+} and Cu^{2+} and (**e**, **f**, **g**, **h**) Pb^{2+}, Cd^{2+} and Cu^{2+}.

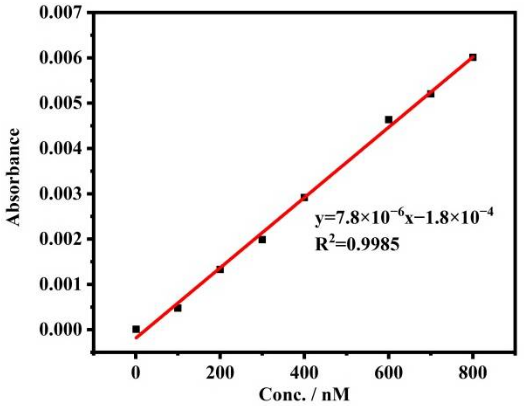

Figure S4. Standard curve of AAS for Pb^{2+}.
